# Adhesion Properties and Stability of Non-Polar Polymers Treated by Air Atmospheric-Pressure Plasma

**DOI:** 10.3390/polym15112443

**Published:** 2023-05-25

**Authors:** Emma Bîrleanu, Ilarion Mihăilă, Ionuț Topală, Cătălin Borcia, Gabriela Borcia

**Affiliations:** 1 Iasi Plasma Advanced Research Center (IPARC), Faculty of Physics, Alexandru Ioan Cuza University of Iasi, Blvd. Carol I No. 11, 700506 Iasi, Romania; 2 Integrated Center of Environmental Science Studies in the North-Eastern Development Region (CERNESIM), Alexandru Ioan Cuza University of Iasi, Blvd. Carol I No. 11, 700506 Iasi, Romania

**Keywords:** atmospheric-pressure plasma, polyethylene, polypropylene, polystyrene, adhesion, contact angle, XPS, AFM

## Abstract

Atmospheric-pressure plasma (APP) has advantages for enhancing the adhesion of polymers and has to provide uniform, efficient treatment, which also limits the recovery effect of treated surfaces. This study investigates the effects of APP treatment on polymers that have no oxygen bonded in their structure and varying crystallinity, aiming to assess the maximum level of modification and the post-treatment stability of non-polar polymers based on their initial structure parameters, including the crystalline–amorphous structure. An APP reactor simulating continuous processing operating in air is employed, and the polymers are analyzed using contact angle measurement, XPS, AFM, and XRD. APP treatment significantly enhances the hydrophilic character of the polymers, with semicrystalline polymers exhibiting adhesion work values of approximately 105 mJ/m^2^ and 110 mJ/m^2^ for 0.5 s and 1.0 s exposure, respectively, while amorphous polymers reach approximately 128 mJ/m^2^. The maximum average oxygen uptake is around 30%. Short treatment times induce the roughening of the semicrystalline polymer surfaces, while the amorphous polymer surfaces become smoother. The polymers exhibit a limit to their modification level, with 0.5 s exposure being optimal for significant surface property changes. The treated surfaces remain remarkably stable, with the contact angle only reverting by a few degrees toward that of the untreated state.

## 1. Introduction

Polymers are a class of ubiquitous materials that are essential in all sectors of activity, including commercial, technological, industrial, and scientific fields. They possess a large range of bulk properties, with particularly versatile mechanical properties resulting from the complex combination of their chemical structure, chain structure, presence of functional groups with different polarities in the main chain and/or as pendant groups, and degree of crystallinity [1]. However, polymer surfaces are generally hydrophobic and intrinsically inert, having poor compatibility with other media. Therefore, many applications of polymers require surface processing to enhance their adhesion properties [2,3,4,5,6,7,8,9].

Among the various methods used for the surface processing of materials, plasma treatment offers great versatility. This is due to its shorter processing durations, production of fewer harmful by-products, and, importantly, ability to modify only the topmost layers of the material, thus preserving the bulk [10,11,12]. Additionally, plasma is recommended for the surface treatment of sensitive materials, such as polymers [13].

The plasma effect on polymers arises from a combination of factors, with the initial step involving the cleaning of the exposed material’s surface. This process breaks the weak chemical bonds and eliminates the small molecules, microscopic rusts, oil layers, oligomers, volatile products, and other contaminants with low molecular weight that typically accumulate on the surface during the manufacturing and storage. As a result, plasma treatment achieves a uniformly clean and active surface, generating radicals [14]. Subsequently, these surface radicals initiate a series of chemical reactions, depending to a large extent on the working gas [14,15]. In the case of treatments sustained in inert gas such as helium or argon, the ensuing surface radicals should theoretically interact with each other, leading to crosslinking. Conversely, when plasma is ignited in a chemically reactive atmosphere such as oxygen or nitrogen, the plasma-induced surface radicals interact with the reactive species present in the plasma, resulting in surface functionalization through the incorporation of oxygen- or nitrogen-containing functionalities [14,15,16,17].

In this general context, the interest of using atmospheric-pressure plasma (APP) is generally acknowledged, due to the lower overall costs associated with this technology and its simplicity of operation for surface processing [18]. In particular, APP working in air may offer a technological solution for imparting adjustable adhesion properties to polymer surfaces, within very short exposure durations.

Nonetheless, two main drawbacks were identified in such applications. Firstly, most studies reported the recovery effect of plasma-treated polymer surfaces, due to the natural tendency of the activated higher energy surface to reach equilibrium [17,18]. Secondly, experimental setups needed to generate a sufficiently high density of plasma active species (O^•^, OH^•^, etc.) and to overcome the limitation of the filamentary discharges commonly observed in atmospheric-pressure air environments. This is necessary to ensure efficient and uniform surface treatment [10].

When using APP for the surface processing of polymers, it is important to consider that the extent of surface modification, polarity enhancement, and post-treatment stability can vary for each piece of plasma reactor equipment and depends on several factors. These factors include treatment parameters, polymer class, chemical structure, presence of functional groups, degree of oxidation in terms of intrinsically structurally bonded oxygen, crystallinity, and surface polarity. Among the many classes of polymers, those that lack functional groups and exhibit a high hydrophobic character, such as polyolefins, can provide a consistent representation of the surface treatment outcomes in a given experimental configuration and of the extent of surface recovery [19,20,21,22,23,24,25,26,27].

Taking this into account, we developed a plasma reactor working in air at atmospheric pressure, based on a dielectric barrier discharge (DBD), which allows the surface treatment of materials on a large exposed area, under conditions simulating continuous processing. A study was conducted to investigate the modification of the surface properties of the selected polymers. In this study, non-polar polymers, such as low-density polyethylene (LDPE), high-density polyethylene (HDPE), ultra-high-density polyethylene (UHMWPE), polypropylene (PP), and polystyrene (PS) were chosen. These polymers do not have oxygen bonded in their structure prior to plasma exposure and exhibit varying degrees of crystallinity. Specifically, the polyolefins (PEs and PP) are semicrystalline, while PS is amorphous, as confirmed by X-ray diffractograms. Such low-energy surfaces allow for the assessment of the efficiency of the treatment in terms of adhesion enhancement and oxygen uptake for very short plasma exposure. Therefore, the plasma exposure effects are also studied for two treatment durations.

The surface of polymers, before and after processing, is analyzed by contact angle measurement, X-ray photoelectron spectroscopy (XPS), and atomic force microscopy (AFM). The stability of the surface properties is assessed by contact angle measurement for 14 days after the treatment. Additionally, X-ray diffractograms (XRD) are utilized to determine the degree of crystallinity of the polymers that exhibit crystalline diffraction peaks.

The results show that plasma exposure as short as 0.5 s provides an efficient and mild surface treatment, resulting in major surface modification, which is uniform for larger areas, without degradation of the material. The treatment leads to a substantial enhancement of the hydrophilic character on all surfaces, which is quantitatively expressed by an increase in adhesion work and surface polarity. Significant oxidation is observed on all the polymers, along with modifications in surface roughness that vary based on the initial surface polarity and the crystalline–amorphous structure characteristics. Importantly, post-treatment ageing, indicated by the loss of the hydrophilic character imparted by the plasma exposure, is limited for all polymers. This demonstrates the very good stability of the treated surfaces.

Our research contributes to the design and development of APP technology for surface processing, providing a very convenient tool for the controlled activation and modification of polymers’ surfaces under conditions that simulate continuous high-speed processing. The selection of commercially important polymers that do not contain bonded oxygen in their intrinsic structure prior to treatment enables the evaluation of the behavior of amorphous polymer structures in comparison to semicrystalline ones. This assessment helps determine the relation between polymer structure parameters, plasma conditions, and surface properties, such as adhesion, polarity, and oxidation, also establishing the achievable limit of surface modification.

Our results are valuable in the broader context of polymer-based materials, which have a diverse range of applications and are closely related to surface wettability and necessitate suitable processing methods that preserve the bulk properties, while also restraining the ageing process, thus allowing for operational stability.

## 2. Materials and Methods

### 2.1. Experimental Setup

The surface treatment is carried out using a plasma reactor working in air at atmospheric pressure, based on a dielectric barrier discharge (DBD), which allows surface treatment of materials, for large exposed area, under conditions simulating continuous processing.

The setup consists of an asymmetrical electrode configuration, with adjustable inter-electrode gap. The high-voltage electrode is a double-blade system, working as an electric field concentrator, 20 cm long and 2 mm wide. The sample to be treated is placed on the ground electrode, consisting of a rectangular metallic plate, 30 cm × 20 cm, covered with dielectric layer (polymer film). The interelectrode gap can be adjusted by vertically sliding the power electrode to adapt to samples of different thickness. During the treatment, a motor stage moves the ground electrode, allowing the materials under treatment to pass through the discharge region between the electrodes with linear controlled speed (between 0.3 ÷ 3 cm/s) and, thus, monitor the actual treatment duration. This arrangement permits the control of very short treatment times (fractions of a second). As shown later, plasma exposure shorter than one second can markedly modify the surface properties of the treated specimen.

The discharge is produced using high-voltage pulses, with duration in the range of hundreds of nanoseconds and with very short rise time (<100 ns). The pulses are generated in an electrical circuit including a trigger signal from a function generator, a high-voltage DC supply (Technix SR Series 30 kV, 20 mA, 600 W), and a solid-state electronic switching device (Behlke HTS 300, 30 kV DC, 30 A peak, rise time <10 ns).

During the present experiments, the HV supply is set to apply positive voltage pulses with 9.5 kV amplitude, 40 μs width, and 900 Hz repetition frequency, generating 5 A peak current intensity and resulting in 1.4 mJ/pulse energy transferred to the discharge.

The plasma exposure effects are also studied for two treatment durations, 0.5 s and 1.0 s.

The discharge working in atmospheric air here is of filamentary type. However, due to the random distribution of microdischarges through the interelectrode zone and the continuous movement of the polymer sample during the treatment, the apparent nonuniformity of the randomly occurring filamentary discharge does not necessarily result in operational drawbacks. Experimental results demonstrate that the resulting treatments exhibit effective surface uniformity across test surfaces measuring approximately 10 cm × 10 cm. Furthermore, no localized damage due to polymer degradation occurs within the tested exposure durations. Additionally, the good stability observed in the modified polymer surfaces indicates the mild treatment conditions offered by this specific DBD arrangement.

### 2.2. Materials

A series of experiments were carried out on various types of 0.05 mm thick commercial polymer films (Goodfellow Ltd., Cambridge, UK), selected because they do not contain oxygen in their structure unit but have varying crystallinity. The films tested are low-density polyethylene (LDPE), high-density polyethylene (HDPE), ultra-high-molecular-weight poly(ethylene) (UHMWPE), polypropylene (PP), and polystyrene (PS). Among these, polyethylene represents a “model” material due to its simple chemical formulation. However, there are structural differences among the various polyethylene types (LDPE, HDPE, and UHMWPE), while PP and PS can be classified as “substituted polyethylene” variants. In the case of PP, it only contains methylenic groups, while PS features an aromatic ring as a pendant group.

This selection aims to assess the limit level of modification and the post-treatment stability of such nonpolar structure polymers, in terms of wettability, adhesion, polarity, and oxygen uptake, as a function of their initial structure parameters, where their characteristic crystalline–amorphous structure is also taken into account.

### 2.3. Surface Analysis

The crystalline structure of the polymer films is investigated by X-ray diffraction (XRD), using a Shimadzu LabX D6000 X-ray diffractometer (Shimadzu, Kyoto, Japan), with Cu-K_α_ X-ray source (λ = 1.54059 Å) in Bragg–Brentano configuration. The samples are scanned in the 2θ = 10°–80° range, at 4°/min scanning rate and 2° grazing incidence. The diffraction patterns show peaks, associated with the diffraction on the crystalline phase, superimposed on an amorphous halo. These patterns are fitted with mixed Gaussian–Lorentzian functions, with mixing ratio > 0.8 and linear-type background subtraction. The degree of crystallinity X_c_ calculated from the ratios of the areas under the crystalline peaks A_c_ and the amorphous halo A_a_, per [28], is

X_c_ = A_c_/(A_c_ + A_a_) (1)

The contact angle measurement is carried out by the sessile drop technique, using an automated system to store the drop images via a digital camera (Optika, 4083.B5) with PC-based control, acquisition, and data processing. The values of the static contact angle presented are the average of at least 10 measured values obtained on the imaged sessile liquid drop profile, with a drop size of 1 μL.

Then, the water adhesion work on treated surfaces is calculated, defined as

W_a_ = γ_L_(1 + cos θ), (2)

where θ is the contact angle, and γ_L_ is the surface tension of the test liquid, water (W) or formamide (F), presented in Table 1.

The relative increase in the adhesion work, defined as

ΔW_a_/W_a_ = (W_a(treated)_ − W_a(untreated)_)/W_a(untreated)_ × 100%, (3)

is also used as control parameter for the plasma-induced effect on the polymer surface.

Furthermore, the values of the components in Table 1 are used to calculate the surface energy (γ_S_) of polymer sample and its polar (γ_S_^p^) and dispersive (γ_S_^d^) components, using the Owens, Wendt, Rabel and Kaelble (OWRK) model [29,30].

Then, the total surface energy of the polymer sample is

γ_S_ = γ_S_^d^ + γ_S_^p^ (4)

and the surface polarity is defined as

γ_S_^p^/γ_S_ = γ_S_^p^/(γ_S_^d^ + γ_S_^p^). (5)

Furthermore, the contact angle of water (WCA) is used to monitor the surface’s ageing post-treatment. The post-treatment measurement of WCA is conducted on material strips (~8 cm × 0.5 cm) cut from a larger piece that has been exposed to plasma (~10 cm × 10 cm). The strips are then stored in sealed boxes, in a dry and cool room, throughout the ageing process.

The surface chemical analysis is performed by XPS, with the XPS spectra recorded on a PHI-Ulvac VersProbe 5000 spectrometer, using the Mg-K_α_ line (h*ν* = 1253.6 eV), at 45° take-off angle. The value of 285.0 eV of the hydrocarbon C1s core level is used as a calibration of the energy scale. The peak envelopes are curve-fitted using mixed Gauss–Lorentz component profiles, with mixing ratio > 0.8 and linear-type background subtraction.

The surface morphology of the samples is analyzed by atomic force microscopy (AFM) using a scanning probe microscope (Solver PRO from NT-MDT, Moskow, Russia), in non-contact mode. In order to allow for quantitative comparison of the surface roughness across the investigated samples, all images are taken during one imaging session using the same cantilever (NSC21 from MikroMasch, with typical curvature radius of 10 nm, nominal spring constant of 19.1 N/m, and free resonant frequency of 227.9 kHz) and the same laser position. Microscope control, data acquisition, and image analysis are performed using Nova software from NT-MDT. The surface morphology is characterized by means of texture parameters, as arithmetic mean roughness R_am_, root mean roughness R_rms_, and maximum roughness R_max_.

## 3. Results and Discussion

### 3.1. XRD Analysis

**Figure 1:**
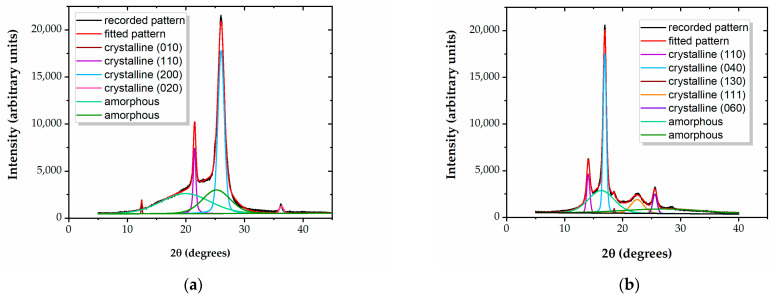
Curve-fitted diffractograms for (**a**) LDPE and (**b**) PP.

Examples of the curve-fitting of diffractograms, allowing for calculations of the degree of crystallinity X_c_, are presented in Figure 1a,b, for the LDPE and PP polymers. The crystalline peaks are assigned based on reference patterns [31,32,33,34,35,36]. Table 2 shows the values of X_c_ for the five tested materials.

It results that the three PEs and the PP films have distinct crystalline–amorphous structures, while PS does not show any diffraction peaks, indicating its completely amorphous nature. This distinction between an amorphous structure and a structure embedding crystalline regions within the amorphous matrix may influence the outcomes of the treatment. It is likely that surface modification through exposure in a reactive environment occurs at a higher rate in amorphous polymer regions, compared to crystalline ones.

### 3.2. Contact Angle Measurement

All the untreated polymer surfaces exhibit a distinct hydrophobic character, with the WCA > 90°, except for PS, which has a WCA = 91.5 ± 0.9°, at the lower limit of the hydrophobic domain. UHMWPE has the highest WCA = 109.0 ± 1.1°, likely attributed to the initial presence of a more hydrophobic surface layer that is commonly formed on commercial polymers during manufacturing processes.

**Figure 2:**
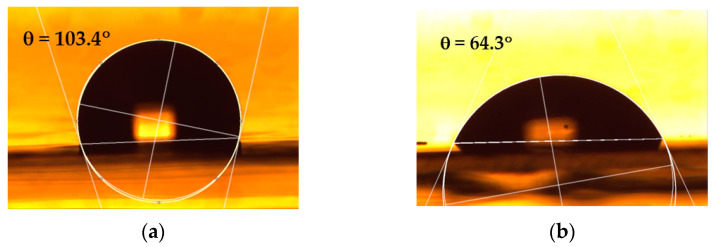
Photographs of water droplets on (**a**) untreated and (**b**) 0.6 s plasma-treated PP, illustrating the measurement of contact angles.

After the plasma treatment, the contact angle of the water (WCA) is observed to undergo changes for all the types of polymer surfaces examined, turning from the hydrophobic character to a hydrophilic one, as shown in Table 3. Figure 2 presents, as an example, photographs of water droplets on both untreated and plasma-treated PP, serving to illustrate the measurement of contact angles.

The steep diminution of the WCA for the treated samples compared to that of the untreated films indicates the strongly increased wettability induced by the plasma exposure for such short treatment times. This behavior can be attributed to the significant surface oxidation that occurs in the discharge, which is further evaluated by XPS analysis and presented later.

The WCA values show that the highest hydrophilic character is observed for the plasma-treated PS, while comparable values are obtained for the other non-polar polymers for the same exposure duration. Moreover, the rate of modification is very high within the first 0.5 s, with only limited additional alteration of the surface wettability during the next 0.5 s of treatment. Interestingly, the modification practically levels out for PS, suggesting that an amorphous polymer structure reaches its limit level of hydrophilicity faster. Anyhow, it can be anticipated that treatments beyond 1 s are in fact too long for further advantageous change in the surface properties, at least for non-polar polymer structures such as those tested here. More extended times may instead lead to the actual degradation of the treated surface.

**Figure 3:**
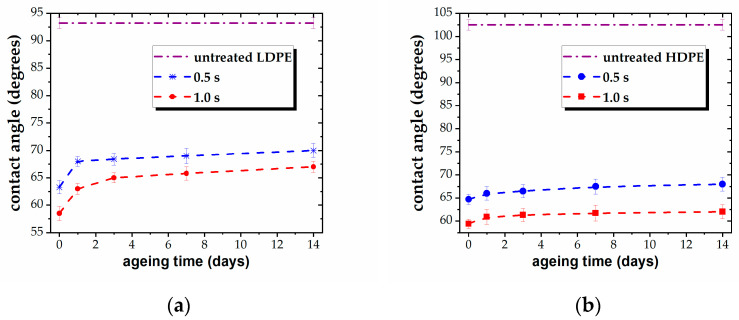

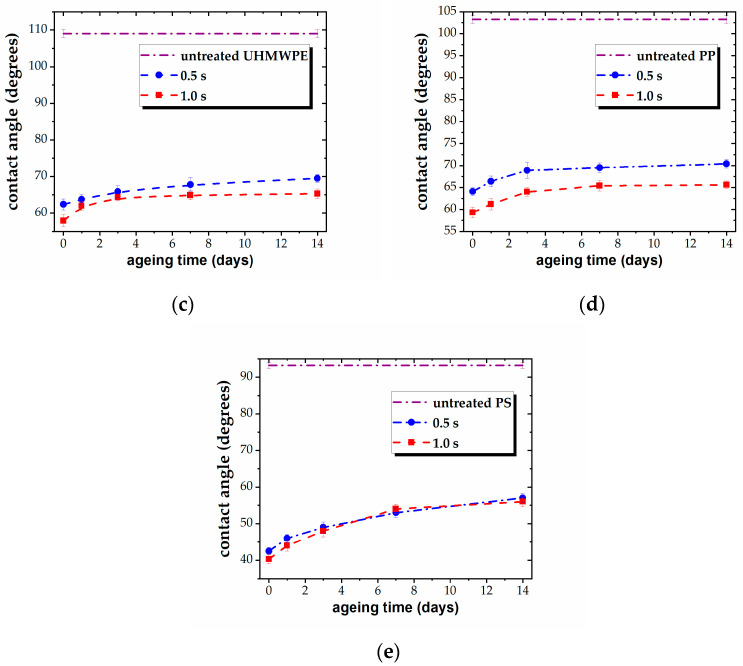
Variation of WCA vs. ageing time, for plasma-treated (**a**) LDPE, (**b**) HDPE, (**c**) UHMWPE, (**d**) PP, and (**e**) PS for different treatment times (0.5 s and 1.0 s).

The values of the WCA offer a suggestive image of the stability of the plasma-exposed surfaces, as the contact angle is very sensitive to any alteration of the surface layer. The results are presented in Figure 3, for all five samples. Thus, the ageing survey shows a low degree of post-treatment recovery for the tested materials. The stability of the three PEs and the PP films is to be emphasized, with the contact angle reverting only by 5°–8° toward that of the untreated state over a period of several days (Figure 3a–d). Specifically, the modified surfaces tend to recover only in the first 2–3 days after treatment, without any further measurable evolution, except in the case of the PS film. The PS surface shows a trend to recover during a longer interval, with the WCA gradually increasing over a period of 14 days after plasma processing, reverting by about 15°. Nonetheless, the strong hydrophilic character of the surface is preserved, and some tendency to level out is to be expected for a longer ageing survey.

The enhancement in the adhesion properties of the plasma-exposed polymers is illustrated by the behavior of the adhesion work of water W_a_, calculated using the WCA values. In this respect, Table 4 summarizes the data on the adhesion work W_a_ and the relative variation of the adhesion work ΔW_a_/W_a_ for plasma-treated polymers.

Thus, the untreated materials can be ranked in terms of adhesion work W_a_ as follows: (UHMWPE) < (HDPE, PP) < (LDPE) < (PS), with all values lower than 72 mJ/m^2^ due to the hydrophobic character of all surfaces. Then, the plasma treatment induces different relative variation ΔW_a_/W_a_ on the tested surfaces, conducting to close values of the adhesion work for the four semicrystalline polymers, ~105 mJ/m^2^ and ~110 mJ/m^2^ for 0.5 s and 1.0 s of exposure, respectively. In contrast, the amorphous PS reaches the highest value of ~128 mJ/m^2^, which is practically similar for both treatment times. It results that the rate of modification of the adhesion work varies for different structures during the first 0.5 s of exposure, ranging between 108% and 234% per second, and subsequently progresses at a much slower rate, which is only ~10% per second for the polyolefin films and 4% per second for PS.

Table 4 shows that the decrease in the adhesion properties of the polymers upon ageing is consistent with the behavior of the WCA, indicating limited surface recovery and good stability. The aged treated surfaces exhibit enhanced adhesion compared to the untreated ones, with higher values for the longer exposure duration, except for the PS sample, for which the values are similar to those for 0.5 s and 1.0 s of exposure.

It also results that polymer surfaces with lower initial adhesion work undergo a more significant modification of their adhesion properties, as indicated by the increase in ΔW_a_/W_a_. The ordering of polymers, in terms of ΔW_a_/W_a_, after plasma exposure, is (UHMWPE) > (HDPE) > (PP) > (LDPE) > (PS), which remains consistent for both (treated) and (treated + aged) samples. It is noteworthy to mention that PS, which exhibits the highest W_a_ from plasma exposure, also displays the highest degree of recovery, suggesting an accelerated tendency of the perturbated surface to lower its energy.

**Figure 4:**
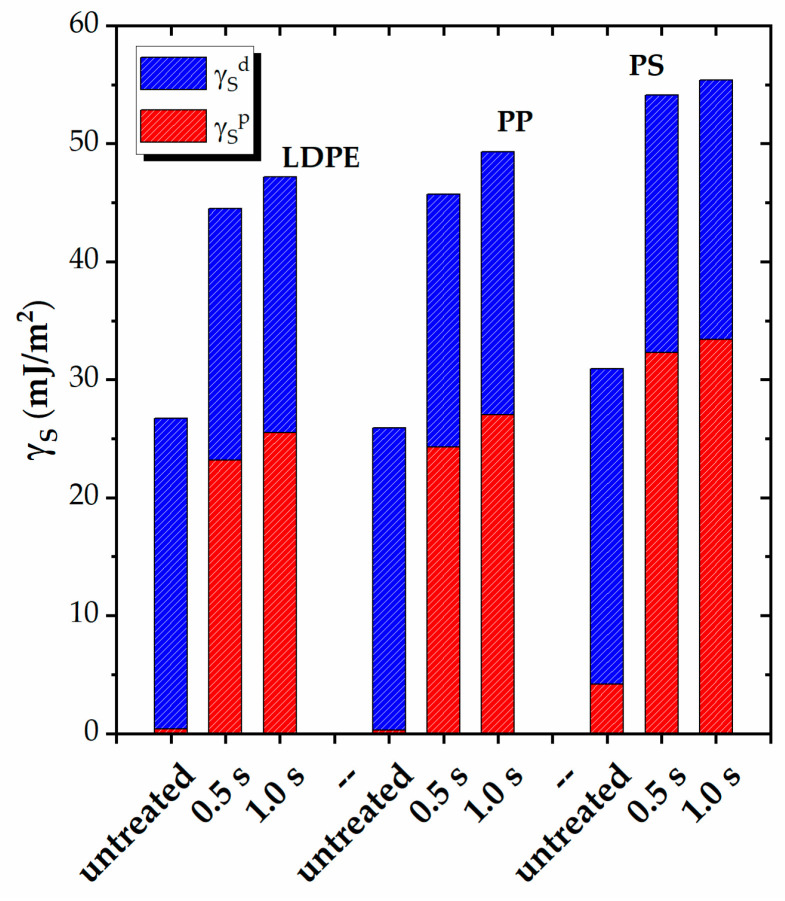
Polar (γ_S_^p^) and dispersive (γ_S_^p^) contributions to the surface energy (γ_S_) of LDPE, PP, and PS before and after plasma treatment for different durations (0.5 s and 1.0 s).

In addition to the water adhesion work, the surface free energy (γ_S_) and its polar (γ_S_^p^) and dispersive (γ_S_^d^) components are calculated for the tested materials, before and after plasma exposure, using the contact angle values for the two test liquids, and the results for the representative polymers are presented in Figure 4. The three PEs exhibit similar behavior in terms of these contributions.

The overall view shows that, prior to plasma treatment, all semicrystalline polymers have practically zero surface energy due to polar contribution, whereas the amorphous PS has a measurable γ_S_^p^ = 4.2 mJ/m^2^. Nonetheless, all polymers have a rather similar low surface energy, with γ_S_ ranging between ~26–30 mJ/m^2^.

Plasma exposure results in an important increase in the polar component of the surface energy for all polymers, with comparable values observed for the PEs and PP, reaching ~25–27 mJ/m^2^. The increase is more pronounced for PS, with a value of ~33 mJ/m^2^. The increase in the surface energy depends, only to a small extent, on the exposure duration, as similar values are obtained for both treatment times. The increase in γ_S_^p^ levels out at ~47–49 mJ/m^2^ for the polyolefins and reaches 55 mJ/m^2^ for PS.

Table 5 presents the surface polarity γ_S_^p^/γ_S_ for all five polymers. The untreated surfaces exhibit nearly zero polarity (~0.01) for the PEs and PP and ~0.14 for PS. However, following plasma exposure, the contribution of the polar groups to the total surface energy becomes significant. The polyolefins demonstrate comparable values of 0.54–0.55, while PS exhibits a higher value of 0.60. This trend indicates the maximum surface polarity attainable by plasma exposure for nonpolar polymer structures.

The higher polarity of plasma-treated PS could be attributed to several factors. Firstly, the untreated PS surface already possesses a hydrophobic character that is at the limit of the hydrophile range, with a contact angle (WCA) of 91.5 ± 0.9°. Additionally, the untreated PS surface exhibits a measurable, albeit low, level of polarity compared to the other four polymers, which have no surface polarity. As a result, the improvement in the wettability and adhesion-related properties of PS is more pronounced compared to that of the other four polymers. Furthermore, the amorphous PS structure may be more susceptible to modification. Highly crystalline polymer structures are likely more resistant to chemical modification, so the amorphous regions are more prone to radical formation, functionalization, and chain scission during plasma processing. However, the relative increase in the polarity γ_S_^p^/γ_S_ of treated surfaces, with respect to the untreated ones, shows very similar values of ~0.5 for all the tested polymers.

### 3.3. Surface Chemical Characterization by XPS

XPS analysis is employed to investigate the chemical structure of the polymer surfaces, taking into account that the reactive species in the discharge is oxygen from the atmospheric air. The XPS spectra are fitted based on reference measurements, and the carbon chemical groups are identified and numbered in increasing order of their binding energy [15,37,38].

All untreated surfaces have the same profile of the carbon C1s spectrum, with the reference hydrocarbon peak C1 at 285.0 eV. Additionally, a minor contribution from a second peak, C2 at 286.5 eV, is observed, which corresponds to the intrinsic low-level oxidized carbons within the hydrocarbon structure and is attributed to carbon atoms singly bonded to oxygen. The atomic elemental composition of the untreated polymers yields a typical oxygen-to-carbon ratio of ~0.04:1, i.e., ~4% carbon bound to oxygen, for all the samples.

The PS sample exhibits an additional characteristic peak at around 291.8 eV (C5), due the low energy *π***-**π* shake-up transitions accompanying the core level ionization for carbons in the aromatic ring. Note that, for PS, the C1 component is representative of both the aliphatic carbons and aryl carbons that are present.

**Figure 5:**
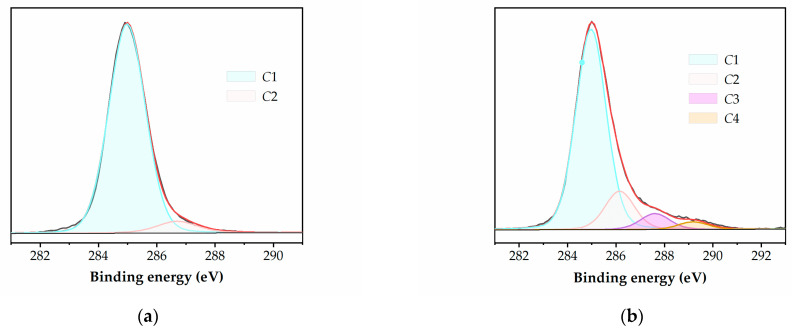
Typical deconvolution of high-resolution C1s XPS spectra for (**a**) untreated and (**b**) 0.5 s plasma-treated LDPE.

The C1s spectra of the plasma-treated polymers show changes in the intensities of the peaks and the appearance of two new components. These components are represented by peaks at 288.0 eV (C3), corresponding to the carbonyl –C=O groups, and 289.0 eV (C4), corresponding to the carboxyl –O–C=O groups, which are formed as a result of plasma exposure (as shown in Table 6). In this respect, an example is presented in Figure 5 for untreated and 0.5 s plasma-treated LDPE.

The same highly oxidized features are identified for all the polymers after plasma treatment, i.e., all three oxidized carbon species mentioned above (C2, C3, and C4) are present in the C1s spectra of the samples subjected to both treatment durations.

Table 7 presents the data for the relative atomic composition of the carbon groups resulting from the deconvolution of the C1s high resolution XPS spectra. The data clearly demonstrate an increase in the surface oxidation of all the tested polymers, indicated by the creation of new oxygen-bonded groups and/or by the addition to the existing ones. This is further emphasized by the increase in the oxidation degree O/C, which represents the ratio between the oxidized and un-oxidized carbon atoms on the surface, also presented in Table 6, and is calculated as

O/C = (C2 + C3 + C4)/C1.(6)

The oxygen uptake onto the treated surfaces is significant for the short 0.5 s exposure, but there is a limited additional increase with prolonged treatment. In case of the XPS measurement, there is a small difference between the values corresponding to the two exposure durations, even for the PS sample, contrarily to the wetting behavior, where the contact angle remains unchanged for the longer exposure. This apparent contradiction can be explained by taking into account the different effective depths of the surface layer analyzed by the two techniques. Contact angle measurement practically refers only to the outermost surface layer, whereas XPS probes ~50 Å, the typical escape depth of the photoelectrons concerned. This shows that although the amorphous structure very quickly reaches its limit level of modification at the surface, some further evolution may arise in the subsurface layers. However, the difference between the values corresponding to the two treatment times shown in Table 7 is within 2–3 atom % percentages.

In addition, the intensity of the C3 and C4 components is lower in PS compared to other polymer structures. This distinctive behavior can be attributed to the unique chemical structures of the respective polymer materials. The main mechanism for surface functionalization is triggered by breaking the C–H and C–C bonds present in the aliphatic chain and in the pendent CH_x_ groups, resulting in the formation of free radicals and new chemical bonds by reactions with the oxygen species activated in the discharge. Due to the increased stability of the aromatic ring relative to the aliphatic backbone in PS, it is less likely to be disturbed by the discharge, which is supported by the absence of noticeable changes in the relative intensity of the polystyrene shake-up’s C5 component.

The maximum oxygen uptake reaches ~34% for the three PEs, ~31% for PP, and ~28% for PS.

The XPS results exhibit a similar trend as the contact angle measurement, confirming that a treatment duration as short as 0.5 s is optimal for achieving substantial surface modification. Prolonged exposure does not conduct any significant further alteration to the surface properties and may instead result in excessive treatment, including the reversal of the oxidation due to the loss of low mass volatile fragments, such as CO or CO_2_, and, eventually, to etching and degradation.

### 3.4. AFM Surface Morphology Analysis

**Figure 6:**
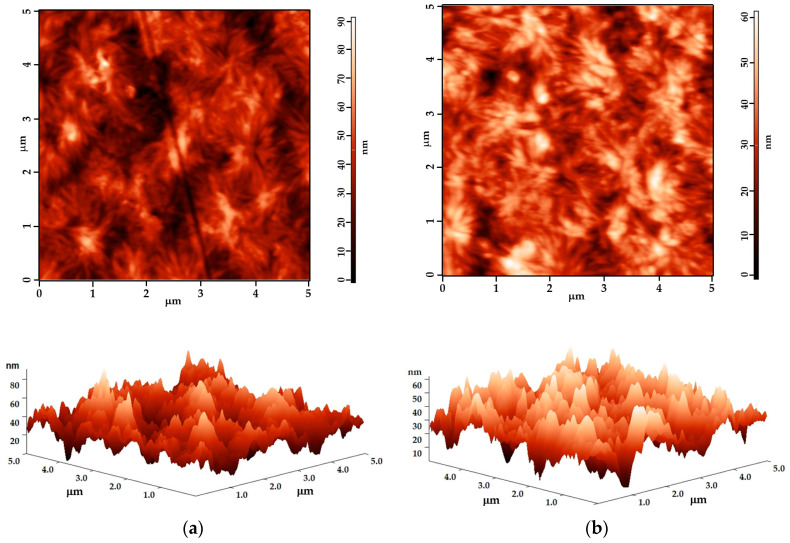
AFM 2D and 3D images (5 μm × 5 μm) for (**a**) untreated PS and (**b**) 1.0 s plasma-treated PS.

The AFM images demonstrate that the surface is not degraded for the tested short exposure durations, indicating that this DBD arrangement offers mild treatment conditions for polymer surfaces. Figure 6 presents examples of the surface topography for both the untreated and treated samples. The surface morphology’s texture parameters for the representative polymers are presented in Table 8.

An analysis reveals that the modification of the average surface roughness on the treated surfaces is minimal, typically only a few nanometers. This outcome is consistent with previous research on various polymers treated with different atmospheric-pressure DBD arrangements, where the maximum variation of R_am_ and R_rms_ was found to be around 3–6 nm [39]. In fact, a significant modification of the roughness could indicate surface degradation caused by the extended treatment. The maximum roughness shows a larger variation, as expected.

Interestingly, the evolution trend varies for different polymer structures. In the case of the three PEs and PP, an increase in surface roughness is observed, which is consistent with most studies [39]. However, for PS, the treated surface is smoother compared to the untreated one. Specifically, the 0.5 s plasma exposure leads to the roughening of the PEs and PP’s surfaces, but there is a tendency to reverse this modification, since the roughness is lower for the 1.0 s treated surfaces than for the 0.5 s treated ones. In contrast, PS consistently shows a trend of smoothening, which is minimal during the first 0.5 s of exposure but becomes measurable for the 1.0 s treatment time.

The difference in behavior may be attributed to the amorphous structure of PS compared to that of the semicrystalline polymers. This suggests that a 0.5 s plasma exposure is indeed optimal for inducing beneficial effects on the treated polymer surfaces. Prolonged treatment is susceptible to conduct a higher ablation rate and the reversal of the surface properties.

## 4. Conclusions

Plasma exposure significantly enhances the hydrophilic character of all surfaces, as evidenced by the increase in the adhesion work, surface polarity, and measurable oxidation on all the polymers. Furthermore, post-treatment ageing reveals a limited loss of the hydrophilic character imparted by the plasma exposure, indicating the very good stability of the treated surfaces.

The rate of modification of the wettability and adhesion-related parameters is most prominent within the first 0.5 s of treatment, with only minor additional changes occurring during the subsequent 0.5 s. These results also suggest that amorphous polymer structures reach their hydrophilicity limit faster. The extent of the adhesion properties modification is more pronounced on polymer surfaces with lower initial adhesion work, and the polymer surface with the highest adhesion work after plasma exposure exhibits the greatest degree of recovery. The contribution of polar groups to the total surface energy becomes significant after treatment, with a comparable increase in the surface polarity for all the tested polymers.

The XPS results align with the contact angle measurement, showing significant oxygen uptake during the short 0.5 s exposure, while any further increase for a prolonged treatment time is minimal. Additionally, the analysis of the surface morphology demonstrates that the surface is not degraded during the tested short exposure durations, with the plasma effect leading to either the roughening or smoothening of the semicrystalline and amorphous polymer structures.

The experimental arrangement used for the plasma exposure provides an efficient and mild surface treatment, with 0.5 s of exposure identified as the optimal amount of time for acquiring significant surface modification. This demonstrates the limit of modification that is achievable by polymer structures in terms of oxygen content and the maximum stress that is tolerated by the surface due to the enhanced polarity.

## Figures and Tables

**Table 1:** Surface tension components of test liquids used for contact angle measurement.

Test Liquid	γ_L_ (mJ/m^2^)	γ_L_^d^ (mJ/m^2^)	γ_L_^p^ (mJ/m^2^)
Water (W)	72.8	21.8	51.0
Formamide (F)	58.2	35.1	23.1

**Table 2:** Degree of crystallinity X_c_ for untreated LDPE, HDPE, UHMWPE, PP, and PS.

Polymer	LDPE	HDPE	UHMWPE	PP	PS
X_c_	0.42 ± 0.02	0.23 ± 0.02	0.26 ± 0.03	0.38 ± 0.03	—

**Table 3:** The contact angle of water (WCA) (°) measured for LDPE, HDPE, UHMWPE, PP, and PS on untreated and plasma-treated surfaces for different treatment times (0.5 s and 1.0 s).

Treatment Time	LDPE	HDPE	UHMWPE	PP	PS
Untreated	100.2 ± 1.0	102.5 ± 1.1	109.0 ± 1.1	103.2 ± 0.9	91.5 ± 0.9
0.5 s	63.3 ± 0.9	64.7 ± 1.1	65.4 ± 1.2	64.1 ± 1.0	42.5 ± 1.4
1.0 s	58.5 ± 1.2	59.4 ± 1.0	57.9 ± 1.3	59.3 ± 1.1	40.3 ± 1.2

**Table 4:** Adhesion work W_a_ (mJ/m^2^) and relative variation of the adhesion work ΔW_a_/W_a_ (%) calculated for LDPE, HDPE, UHMWPE, PP, and PS on untreated surfaces, plasma-treated surfaces for different treatment times (0.5 s and 1.0 s), and aged plasma-treated surfaces.

		LDPE	HDPE	UHMWPE	PP	PS
Untreated	W_a_ (mJ/m^2^)	59.9	57.0	49.0	56.2	68.7
0.5 s	W_a_ (mJ/m^2^)	105.5	103.9	106.5	104.6	126.5
	ΔW_a_/W_a_ (%)	76%	82%	117%	86%	84%
Aged 14 days	ΔW_a_/W_a_ (%)	63%	75%	101%	73%	64%
1.0 s	W_a_ (mJ/m^2^)	110.8	109.8	111.5	110.0	128.3
	ΔW_a_/W_a_ (%)	85%	93%	127%	96%	86%
Aged 14 days	ΔW_a_/W_a_ (%)	69%	88%	110%	83%	65%

**Table 5:** Surface polarity γ_S_^p^/γ_S_ calculated for LDPE, HDPE, UHMWPE, PP, and PS on untreated and plasma-treated surfaces for different treatment times (0.5 s and 1.0 s).

Treatment Time	LDPE	HDPE	UHMWPE	PP	PS
untreated	0.014	0.011	0.008	0.011	0.136
0.5 s	0.521	0.544	0.540	0.531	0.597
1.0 s	0.540	0.556	0.554	0.548	0.602

**Table 6:** Binding energies of carbon functional groups in the C1s fitted spectra for plasma-treated LDPE, HDPE, UHMWPE, PP, and PS.

Functional Groups	Assignment		Binding Energy (eV)
	PEs/PP	PS	
carbon-hydrogen –C–C–, –C–H	C1	C1	285.0
carbon–oxygen –C–O–	C2	C2	286.5 ± 0.2
carbonyl –C=O	C3	C3	288.0 ± 0.2
carboxyl –O–C=O	C4	C4	289.0 ± 0.2
*π*-π* shake-up	-	C5	~291.8

**Table 7:** Atomic composition of the carbon species C1s (in atom %, ±0.5 at. %) and oxidation degree O/C for LDPE, HDPE, UHMWPE, PP, and PS on untreated and plasma treated surfaces for different treatment times (0.5 s and 1.0 s).

	LDPE			HDPE			UHMWPE			PP			PS		
	**0 s**	**0.5 s**	**1.0 s**	**0 s**	**0.5 s**	**1.0 s**	**0 s**	**0.5 s**	**1.0 s**	**0 s**	**0.5 s**	**1.0 s**	**0 s**	**0.5 s**	**1.0 s**
C1	96.2	76.4	74.2	95.8	77.0	75.5	96.5	76.7	75.0	95.4	77.6	76.1	94.8	79.3	77.8
C2	3.8	14.5	15.0	4.2	13.9	14.4	3.5	14.1	14.8	4.6	13.6	13.9	5.2	18.3	19.4
C3	—	6.0	6.9	—	6.2	6.6	—	6.4	6.8	—	5.4	6.2	—	1.3	1.6
C4	—	3.1	3.9	—	2.9	3.5	—	2.8	3.4	—	3.4	3.8	—	1.1	1.2
O/C	0.04	0.31	0.35	0.04	0.30	0.32	0.03	0.30	0.33	0.05	0.28	0.31	0.05	0.26	0.28

**Table 8:** Roughness values R_am_, R_rms_, and R_max_ (±0.5 nm) for LDPE, PP, and PS on untreated and plasma-treated surfaces for different treatment times (0.5 s and 1.0 s).

	LDPE			PP			PS		
	0 s	0.5 s	1.0 s	0 s	0.5 s	1.0 s	0 s	0.5 s	1.0 s
R_am_ (nm)	9.7	12.4	11.5	8.4	13.5	12.7	7.7	7.5	5.8
R_rms_ (nm)	12.4	16.7	13.9	12.4	17.1	15.9	9.8	9.4	7.3
R_max_ (nm)	84	115	108	82	120	116	77	70	60

## Data Availability

Not applicable.
